# The Multidirectional Influence of Feed-Borne Deoxynivalenol and Zearalenone on Animal Health

**DOI:** 10.3390/toxins15070419

**Published:** 2023-06-28

**Authors:** Maciej T. Gajęcki, Magdalena Gajęcka

**Affiliations:** Department of Veterinary Prevention and Feed Hygiene, Faculty of Veterinary Medicine, University of Warmia and Mazury in Olsztyn, Oczapowskiego 13, 10-718 Olsztyn, Poland; gajecki@uwm.edu.pl

Mycotoxins are secondary fungal metabolites which pose a significant threat for global food and feed security [1], due to their adverse effects on human and animal health [2], high chemical stability and ubiquitous presence [3]. The simultaneous exposure to several mycotoxins produced by the same or different fungal species exacerbates the risk of food and feed toxicity [4,5]. According to research, plant materials are often contaminated with both DON and ZEN, and the health risks associated with simultaneous exposure to both mycotoxins constitute an interesting topic of study [6,7]. 

Present in plant material, DON and ZEN belong to a large group of fusarium mycotoxins [8] which are produced by various fungal species, including *Fusarium*, *Myrothecium*, *Cephalosporium*, *Verticimonosporium* and *Stachybotrys* [3]. To date, the following mechanisms of toxicity of these mycotoxins have been identified in cells or proteins: (i) DON binds to the 60S ribosome subunit at the molecular level and induces ribotoxic stress, which activates protein kinase and, consequently, inhibits protein synthesis, and provokes endoplasmic reticulum stress [9], cell signalling, cell differentiation, cell proliferation and cell death [5,10]; (ii) ZEN [11] exerts toxic effects by binding to and activating both ERs, disrupting the cell cycle and inducing DNA fragmentation, which leads to the production of micronuclei and chromosomal aberrations [4,5,10]. 

Mycotoxicosis are ambiguous subclinical disorders that affect livestock herds [12,13]. These disorders can be caused by the chronic impairment of general bodily functions [14] or the increased susceptibility of specific tissues [15,16]. Acute poisoning and severe mycotoxicosis are less frequently reported. Complex toxicological interactions (additive effects, synergism, potentiation, and antagonism between mycotoxins) and the dose absorbed [17] undoubtedly affect health and reproductive processes [18]. Depending on the absorbed dose, the interactions between co-occurring mycotoxins or between mycotoxins and specific tissues in mammals [19,20,21] may require further investigation and risk assessments [22] based on an analysis of the biological activity of individual mycotoxins [12,16,17]. 

Low-dose exposure usually leads to subclinical states characterized by specific effects which are manifested by (i) the modulation of feminization processes in sexually immature gilts (which inhibits the somatic development of reproductive system tissues); (ii) disruptions in the neuroendocrine coordination of reproductive competence [14,15,16,19,23]; (iii) the balance between intestinal cells and the expression of selected genes encoding enzymes that participate in biotransformation processes in the large intestine [24]; and (iv) flexible, adaptive responses to low mycotoxin doses. Zearalenone (ZEN) and deoxynivalenol (DON) also induce non-specific effects that do not always decrease the feed conversion efficiency [20,21] and do not lead to a deterioration in the animals’ overall health [25]. In addition, some mycotoxins, including DON, inhibit the activity of biologically active substances [18]. Therefore, their effects are determined by the dose and the duration of exposure.

According to the literature, systems for monitoring mycotoxins in animals should not be based solely on the results of blood tests [12,15]. A solution that delivers reliable results has been proposed in in one of the published studies [12]. The cited study demonstrated that blood samples from clinically healthy cows and/or cows with subclinical symptoms of ZEN mycotoxicosis should be collected from the caudal vein medium (prehepatic blood vessel) for toxicological tests. Samples collected from this site increase the probability that subclinical ZEN mycotoxicosis will be reliably diagnosed.

The monitoring system is a highly practical tool for identifying contaminated herds in the field and for evaluating the impact of chronic exposure on herd health and productivity. Other matrices, such as urine, can also be effectively used for this purpose [16].

However, preventive measures involving other matrices, such as feed materials (primary and partially processed products), are always preferable [13]. This type of monitoring relies on biosensor technologies that offer fast, highly selective, and highly sensitive detection methods, require minimal sample pre-treatment, and reduce reagent consumption. This article reviews recent advances in the development of biosensors for the quantification of DON and ZEN in cereals and feed, which substantially contribute to feed safety. 

The articles published in the Special Issue entitled “Influence of Deoxynivalenol and Zearalenone in Feed on Animal Health” document the in vivo effects of low or very low doses of ZEN and its metabolites on mammals. These effects can vary, and remain insufficiently investigated. The above observations could also apply to other mycotoxins, including DON. Sexually immature gilts respond differently to mycotoxins. The ratio of α-ZEL (alpha-zearalenol) to β-ZEL (beta-zearalenol), where β-ZEL is the predominant compound, could be one of the first biomarkers of mycotoxin contamination. The value of this parameter is different in other age groups. This effect is ambiguous because β-ZEL contributes to a minor increase in body weight, while slowing down the sexual maturation of immature gilts. Initially, ZEN levels are very low, and metabolites are not detected in the blood serum (especially at the MABEL dose), which confirms that gilts have a high physiological demand for exogenous estrogen-like substances. These substances are fully utilized by immature gilts. Exposure to higher mycotoxin doses generates “free ZEN”, which plays different, not always positive roles. The concentrations of estradiol and “free ZEN” increase proportionally to the ZEN dose, which decreases progesterone and testosterone levels [26]. At the same time, the metabolic profile points to a greater loss of energy and protein (stimulation), which suggests that feed is used more efficiently (weight gain) and that mycotoxins are highly involved in biotransformation and detoxification processes. Changes in the metabolic profile fluctuate over time. In the initial period of exposure, metabolic activity is relatively high, which could also be attributed to the compensatory effect. In successive periods, energy-intensive processes initiate adaptive mechanisms. These mechanisms could also be triggered by the increasing involvement of β-ZEL in the final biotransformation process. 

The results of selected diagnostic tests could be used as biomarkers of prolonged low-dose ZEN mycotoxicosis in sexually immature gilts in precision veterinary medicine.

The question that arises is whether cereal grains contaminated with such low doses of ZEN and DON should be detoxified or eliminated from feed production. The results of the study suggest that such low mycotoxin doses should be tolerated due to their potentially stimulating effects on sexually immature gilts in commercial farms.
